# Inhibition of Both Protease and Helicase Activities of Hepatitis C Virus NS3 by an Ethyl Acetate Extract of Marine Sponge *Amphimedon* sp

**DOI:** 10.1371/journal.pone.0048685

**Published:** 2012-11-07

**Authors:** Yuusuke Fujimoto, Kazi Abdus Salam, Atsushi Furuta, Yasuyoshi Matsuda, Osamu Fujita, Hidenori Tani, Masanori Ikeda, Nobuyuki Kato, Naoya Sakamoto, Shinya Maekawa, Nobuyuki Enomoto, Nicole J. de Voogd, Masamichi Nakakoshi, Masayoshi Tsubuki, Yuji Sekiguchi, Satoshi Tsuneda, Nobuyoshi Akimitsu, Naohiro Noda, Atsuya Yamashita, Junichi Tanaka, Kohji Moriishi

**Affiliations:** 1 Department of Microbiology, Division of Medicine, Graduate School of Medicine and Engineering, University of Yamanashi, Yamanashi, Japan; 2 Radioisotope Center, The University of Tokyo, Tokyo, Japan; 3 Biomedical Research Institute, National Institute of Advanced Industrial Science and Technology, Ibaraki, Japan; 4 Department of Life Science and Medical Bioscience, Waseda University, Tokyo, Japan; 5 Research Institute for Environmental Management Technology, National Institute of Advanced Industrial Science and Technology, Ibaraki, Japan; 6 Department of Tumor Virology, Okayama University Graduate School of Medicine, Dentistry, and Pharmaceutical Sciences, Okayama, Japan; 7 Department of Gastroenterology and Hepatology, Hokkaido University Graduate School of Medicine, Sapporo, Japan; 8 First Department of Internal Medicine, Faculty of Medicine, University of Yamanashi, Yamanashi, Japan; 9 Netherlands Centre for Biodiversity Naturalis, Leiden, The Netherlands; 10 Institute of Medical Chemistry, Hoshi University, Tokyo, Japan; 11 Department of Chemistry, Biology and Marine Science, University of the Ryukyus, Okinawa, Japan; Osaka University Graduate School of Medicine, Japan

## Abstract

Combination therapy with ribavirin, interferon, and viral protease inhibitors could be expected to elicit a high level of sustained virologic response in patients infected with hepatitis C virus (HCV). However, several severe side effects of this combination therapy have been encountered in clinical trials. In order to develop more effective and safer anti-HCV compounds, we employed the replicon systems derived from several strains of HCV to screen 84 extracts from 54 organisms that were gathered from the sea surrounding Okinawa Prefecture, Japan. The ethyl acetate-soluble extract that was prepared from marine sponge *Amphimedon* sp. showed the highest inhibitory effect on viral replication, with EC_50_ values of 1.5 and 24.9 µg/ml in sub-genomic replicon cell lines derived from genotypes 1b and 2a, respectively. But the extract had no effect on interferon-inducing signaling or cytotoxicity. Treatment with the extract inhibited virus production by 30% relative to the control in the JFH1-Huh7 cell culture system. The *in vitro* enzymological assays revealed that treatment with the extract suppressed both helicase and protease activities of NS3 with IC_50_ values of 18.9 and 10.9 µg/ml, respectively. Treatment with the extract of *Amphimedon* sp. inhibited RNA-binding ability but not ATPase activity. These results suggest that the novel compound(s) included in *Amphimedon* sp. can target the protease and helicase activities of HCV NS3.

## Introduction

Hepatitis C virus (HCV) is an enveloped RNA virus of the genus *Hepacivirus* of the *Flaviviridae* family. More than 170 million patients persistently infected with HCV have been reported worldwide, leading to liver diseases including steatosis, cirrhosis, and hepatocellular carcinoma [1], [2]. The genome of HCV is characterized as a single positive-strand RNA with a nucleotide length of 9.6 kb, flanked by 5′ and 3′-untranslated regions (UTRs). The genomic RNA encodes a large polyprotein consisting of approximately 3,000 amino acids [3], which is translated under the control of an internal ribosome entry site (IRES) located within the 5′-UTR of the genomic RNA [4]. The translated polyprotein is cleaved by host and viral proteases, resulting in 10 mature viral proteins [3]. The structural proteins, consisting of core, E1, and E2, are located in the N-terminal quarter of the polyprotein, followed by viroporin p7, which has not yet been classified into a structural or nonstructural protein. Further cleavage of the remaining portion by viral proteases produces six nonstructural proteins–NS2, NS3, NS4A, NS4B, NS5A, and NS5B–which form a viral replication complex with various host factors. The viral protease NS2 cleaves its own C-terminal between NS2 and NS3. After that, NS3 cleaves the C-terminal ends of NS3 and NS4A and then forms a complex with NS4A. The NS3/4A complex becomes a fully active form to cleave the C-terminal parts of the polyprotein, including nonstructural proteins. NS3 also possesses RNA helicase activity to unwind the double-stranded RNA during the synthesis of genomic RNA [5], [6].

Although the previous standard therapy, combining pegylated interferon with ribavirin, was effective in only about half of patients infected with genotype 1, the most common genotype worldwide [7]–[9], recent biotechnological advances have led to the development of a novel therapy using anti-HCV agents that directly target HCV proteins or host factors required for HCV replication and have improved the sustained virologic response (SVR) [10]–[12]. Telaprevir and boceprevir, which are categorized as advanced NS3/4A protease inhibitors, were recently approved for the treatment of chronic hepatitis C patients infected with genotype 1 [13], [14]. The triple combination therapy with pegylated interferon, ribavirin, and telaprevir improved SVR by 77% in patients infected with genotype 1 [15]. However, this therapy exhibits side effects including rash, severe cutaneous eruption, influenza-like symptoms, cytopenias, depression, and anemia [7], [16], [17]. Furthermore, the possibility of the emergence of drug-resistant viruses is a serious problem with therapies that use antiviral compounds [18], [19].

Recent technical advances in the determination of molecular structures and the synthesis of chemical compounds have led to the development of various drugs based on natural products, especially drugs identified from terrestrial plants and microbes [20]–[22]. Marine organisms, including plants and animals, were recently established as representative of a natural resource library for drug development. Potent biological activity is often found in products isolated from marine organisms because of their novel molecular structures [23], [24]. Trabectedin (Yondelis), cytarabine (Ara-C), and eribulin (Halaven), which are known as antitumor drugs, were developed from compounds found in marine organisms [25].

In this study, we screened 84 extracts prepared from 54 marine organisms by using replicon cell lines derived from HCV genotype 1b and attempted to identify the extract that inhibits HCV RNA replication. A marine organism may produce anti-HCV agent(s) that could inhibit the protease and helicase activities of NS3.

## Results

### Effect of the Extract from Marine Sponge and Tunicate on HCV Replication

We prepared methanol (MeOH)- and ethyl acetate (EtOAc)-soluble extracts from 54 marine organisms in order to test which of these extracts could best suppress HCV replication. Each extract was added at 25 µg/ml to the culture supernatant of HCV replicon cell lines derived from O and Con1 strains of genotype 1b, which produce the luciferase/neomycin hybrid protein depending on RNA replication. Luciferase activity and cell viability were measured 72 h after treatment with the extracts (Table 1). The extracts exhibiting more than 85% cell viability and lower than 15% luciferase activity were selected as arbitrary candidates for the extract including anti-HCV compounds. The EtOAc-extract prepared from sample C-29 (C-29EA) was selected as a candidate in both cell lines. Thus, the anti-HCV activity of extract C-29EA was tested.

**Table 1:** Effect of marine organism extracts on HCV replication and cell viability.

No.	Sample	Luciferase activity(% of control)		Cell viability(% of control)		Phylum	Specimen	Extract	Site
		O	Con1	O	Con1				
1	A-1	10	111	105	104	Sponge	*Unidentified*	MeOH	A
2	A-2	82	209	91	132	Soft coral	*Briareum*	MeOH	A
3	A-3	87	177	54	110	Tunicate	*unidentified*	MeOH	A
4	A-4	82	186	84	100	Sponge	*Liosina*	MeOH	A
5	B-5	110	165	86	110	Sponge	*unidentified*	MeOH	B
6	B-6	70	149	103	119	Sponge	*Xestospongia*	MeOH	B
7	B-7	89	191	111	144	Sponge	*Epipolasis*	MeOH	B
8	B-8	89	182	115	132	Sponge	*unidentified*	MeOH	B
9	B-9	57	72	92	124	Sponge	*Strongylophora*	MeOH	B
10	B-10	106	182	73	96	Sponge	*Stylotella aurantium*	MeOH	B
11	C-12	96	162	114	98	Sponge	*Epipolasis*	MeOH	B
12	C-13	123	141	91	103	Sponge	*unidentified*	MeOH	B
13	C-14	89	175	77	100	Sponge	*Hippospongia*	MeOH	B
14	C-16	80	177	108	88	Sponge	*unidentified*	MeOH	B
15	C-18	119	170	93	94	Sponge	*unidentified*	MeOH	B
16	C-19	0	0	0	4	Sponge	*unidentified*	MeOH	B
17	C-20	101	158	61	106	Sponge	*Xestospongia testudinaria*	MeOH	B
18	C-21	85	161	83	102	Sponge	*unidentified*	MeOH	B
19	C-22	109	88	38	89	Sponge	*unidentified*	MeOH	B
20	C-23	94	156	32	90	Sponge	*unidentified*	MeOH	B
21	C-24	118	86	42	94	Sponge	*Theonella*	MeOH	B
22	C-25	82	111	91	106	Sponge	*unidentified*	MeOH	B
23	C-27	0	0	15	2	Sponge	*unidentified*	MeOH	B
24	C-28	90	166	30	90	Sponge	*Petrosia*	MeOH	B
25	C-29	65	151	29	101	Sponge	*Amphimedon*	MeOH	B
26	D-31	81	127	55	91	Tunicate	*unidentified*	MeOH	C
27	D-32	80	141	47	93	Sponge	*unidentified*	MeOH	C
28	D-33	88	153	72	90	Gorgonian	*Junceella fragilis*	MeOH	C
29	E-35	114	156	40	118	Sponge	*Phyllospongia sp.*	MeOH	C
30	E-36	80	125	69	116	Tunicate	*Didemnum molle*	MeOH	C
31	E-37	88	129	54	108	Sponge	*Xestospongia sp.*	MeOH	C
32	E-38	70	153	35	112	Sponge	*unidentified*	MeOH	C
33	F-40	119	170	38	104	Sponge	*unidentified*	MeOH	C
34	F-41	88	166	48	101	Soft coral	*unidentified*	MeOH	C
35	G-42	113	157	31	126	Sponge	*unidentified*	MeOH	D
36	H-43	83	0	39	5	Sponge	*unidentified*	MeOH	D
37	J-44	62	183	27	105	Sponge	*Cinachyra*	MeOH	D
38	J-45	96	140	47	103	Sponge	*Liosina*	MeOH	D
39	J-46	83	149	77	102	Sponge	*unidentified*	MeOH	D
40	J-47	94	37	40	111	Sponge	*unidentified*	MeOH	D
41	J-48	24	16	53	70	Sponge	*Stylotella*	MeOH	D
42	J-49	78	123	55	105	Sponge	*unidentified*	MeOH	D
43	J-50	93	138	51	108	Sponge	*unidentified*	MeOH	D
44	J-51	103	73	41	115	Sponge	*unidentified*	MeOH	D
45	J-52	162	237	113	131	Sponge	*unidentified*	MeOH	D
46	J-53	51	90	93	122	Tunicate	*Didemnum*	MeOH	D
47	J-54	42	90	113	124	Sponge	*unidentified*	MeOH	D
48	J-55	88	133	131	110	Jellyfish	*unidentified*	MeOH	D
49	J-56	28	51	113	103	Sponge	*unidentified*	MeOH	D
50	J-57	8	63	94	85	Tunicate	*Pseudodistoma kanoko*	MeOH	D
51	J-58	0	2	48	65	Sponge	*unidentified*	MeOH	D
52	J-59	0	2	45	71	Sponge	*unidentified*	MeOH	D
53	J-60	98	134	122	95	Annelid	*unidentified*	MeOH	D
54	A-2	0	1	6	15	Soft coral	*Briareum*	EtOAc	A
55	A-3	0	0	6	9	Tunicate	*unidentified*	EtOAc	A
56	A-4	22	36	74	76	Sponge	*Liosina*	EtOAc	A
57	B-5	33	107	69	93	Sponge	*unidentified*	EtOAc	B
58	B-6	0	0	5	8	Sponge	*Xestospongia*	EtOAc	B
59	B-7	0	0	5	9	Sponge	*Epipolasis*	EtOAc	B
60	B-8	0	0	2	46	Sponge	*unidentified*	EtOAc	B
61	B-9	0	0	8	14	Sponge	*Strongylophora*	EtOAc	B
62	B-10	0	0	3	8	Sponge	*Stylotella aurantium*	EtOAc	B
63	C-12	0	0	4	14	Sponge	*Epipolasis*	EtOAc	B
64	C-13	0	0	4	5	Sponge	*unidentified*	EtOAc	B
65	C-14	48	119	82	102	Sponge	*Hippospongia*	EtOAc	B
66	C-15	0	0	8	11	Sponge	*unidentified*	EtOAc	B
67	C-18	0	0	4	3	Sponge	*unidentified*	EtOAc	B
68	C-19	23	76	63	109	Sponge	*unidentified*	EtOAc	B
69	C-20	34	32	63	112	Sponge	*Xestospongia testudinaria*	EtOAc	B
70	C-21	1	0	52	12	Sponge	*unidentified*	EtOAc	B
71	C-22	76	34	74	110	Sponge	*unidentified*	EtOAc	B
72	C-24	0	0	20	7	Sponge	*Theonella*	EtOAc	B
73	C-26	41	43	80	110	Sponge	*unidentified*	EtOAc	B
74	C-27	1	0	35	40	Sponge	*unidentified*	EtOAc	B
75	C-28	68	62	82	115	Sponge	*Petrosia*	EtOAc	B
76	C-29	10	11	93	88	Sponge	*Amphimedon*	EtOAc	B
77	D-31	20	71	85	120	Tunicate	*Eudistoma*	EtOAc	C
78	D-33	0	0	5	7	Gorgonian	*Junceella fragilis*	EtOAc	C
79	E-35	0	0	4	5	Sponge	*Phyllospongia sp.*	EtOAc	C
80	E-36	71	83	75	100	Tunicate	*Didemnum molle*	EtOAc	C
81	F-40	72	110	87	130	Sponge	*unidentified*	EtOAc	C
82	F-41	8	33	73	104	Soft coral	*unidentified*	EtOAc	C
83	H-43	0	197	4	119	Sponge	*unidentified*	EtOAc	D
84	J-46	113	58	103	126	Sponge	*unidentified*	EtOAc	D

There are a total of 54 marine organisms, while 84 extracts were prepared from them with ethyl acetate and/or methanol. Aragusuku, Iriomote, Kohama, and Ishigaki islands are indicated by A, B, C, and D, respectively, in the collection-site column?(right end). EtOAc: Ethyl acetate; MeOH: Methanol.

**Figure 1:**
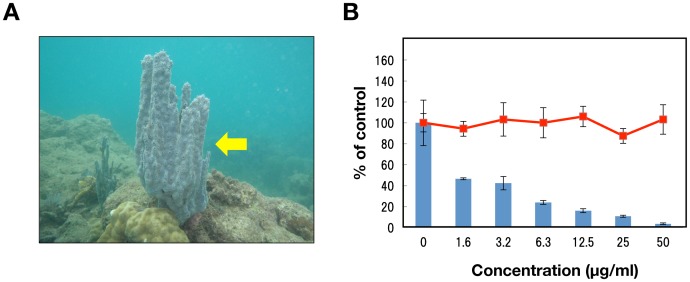
Effect of the extract prepared from a marine sponge on viral replication in the replicon cell line derived from viral genotype 1b. (A) *Amphimedon* sp. belongs to a marine sponge. The ethyl acetate fraction prepared from the marine organism was designated C-29EA in this study. (B) The Huh7 cell line, including the subgenomic replicon RNA of genotype 1b strain Con1, was incubated in medium containing various concentrations of C-29EA or DMSO (0). Luciferase and cytotoxicity assays were carried out as described in Materials and Methods. Error bars indicate standard deviation. The data represent three independent experiments.

**Figure 2:**
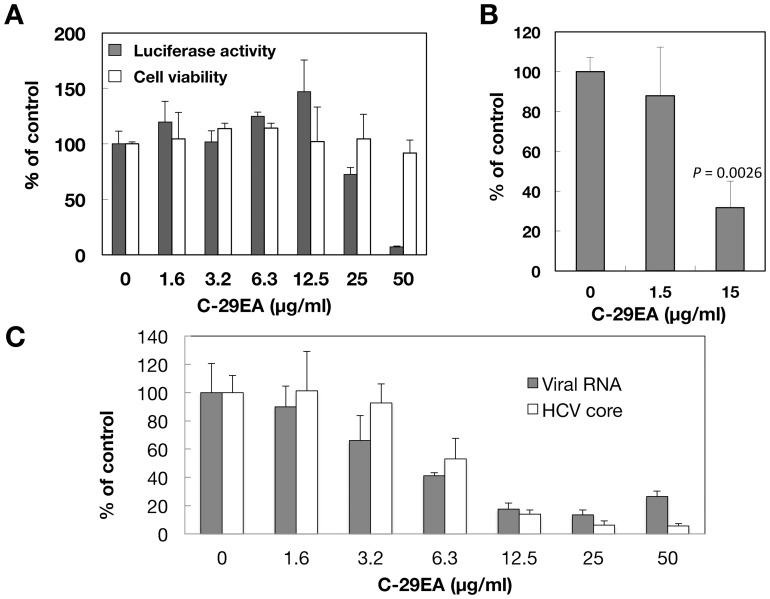
Effect of C-29EA extract on viral replication in the replicon cell line derived from viral genotype 2a. (A) The Huh7 cell line, including the subgenomic replicon RNA of genotype 2a strain JFH1, was incubated in medium containing various concentrations of C-29EA or DMSO (0). Luciferase and cytotoxicity assays were carried out as described in Materials and Methods. (B) The Huh7 OK1 cell line infected with HCVcc JFH1 was incubated with various concentrations of C-29EA or DMSO (0). The virus titers were determined by a focus-forming assay. The significance of differences in the means was determined by Student’s *t*-test. (C) Amounts of viral RNA and core protein were estimated by qRT-PCR and ELISA, respectively. Error bars indicate standard deviation. The data represent three independent experiments. Treatment with DMSO corresponds to ‘0′.

The EtOAc-soluble extract C-29EA was prepared from the marine sponge *Amphimedon* sp. (Fig. 1A), which inhabits the sea surrounding Okinawa Prefecture, Japan. HCV replication was inhibited in a dose-dependent manner but did not exhibit cytotoxicity when replicon cells were treated with C-29EA (Fig. 1 B). The extract C-29EA exhibited EC_50_ values of 1.5 µg/ml (Table 2). Furthermore, treatment with C-29EA suppressed the HCV replication derived from the genotype 2a strain JFH1 with an EC_50_ of 24.9 µg/ml, irrespective of cell viability (Fig. 2A and Table 2). Extract C-29EA also inhibited the production of infectious viral particles, viral RNA, and core protein from JFH1-infected cells in the supernatant (Fig. 2B and C). These results suggest that the marine sponge *Amphimedon* sp. possesses anti-HCV agents.

**Table 2:** Effect of C29EA on HCV replication.

HCV strain (genotype)	EC_50_ (µg/ml)^a^	CC_50_ (µg/ml)^b^	SI^c^
Con 1 (1b)	1.5	>50	>33.3
JFH1 (2a)	24.9	>50	>2.3

a : Fifty percent effective concentration based on the inhibition of HCV replication.

b : Fifty percent cytotoxicity concentration based on the reduction of cell viability.

c : SI, selectivity index (CC_50_/EC_50_).

### Effect of Extract C-29EA on IRES-dependent Translation

**Figure 3:**
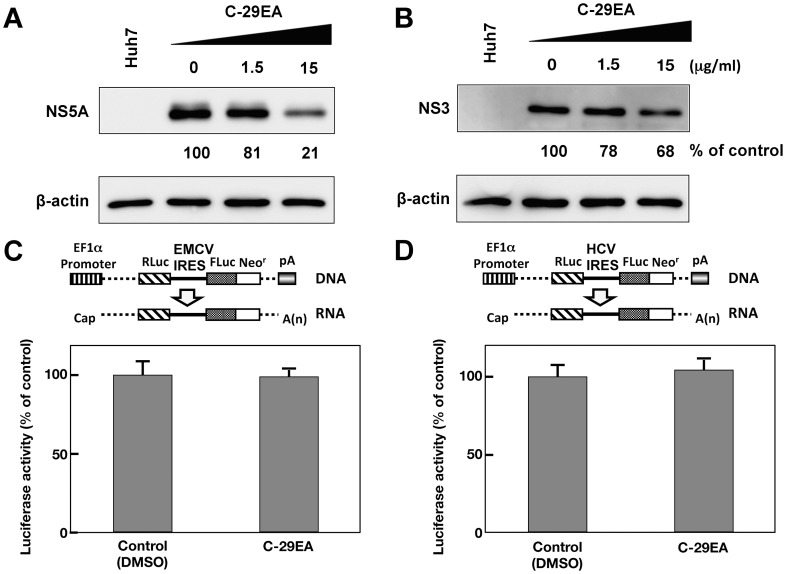
Effect of C-29EA on expression of viral proteins in replicon cell lines. The Huh7 replicon cell lines derived from genotype 1b (A) and 2a (B) were incubated with C-29EA at 37°C for 72 h. The treated cells were harvested and then subjected to Western blotting. Treatment with DMSO corresponds to ‘0′. The bicistronic gene is transcribed under the control of the elongation factor 1α (EF1α) promoter. The upstream cistron encoding *Renilla* luciferase (RLuc) is translated by a cap-dependent mechanism. The downstream cistron encodes the fusion protein (Feo), which consists of the firefly luciferase (Fluc) and neomycin phosphotransferase (Neo^r^), and is translated under the control of the EMCV IRES (C) or HCV IRES (D). The Huh7 cell line transfected with the plasmid (each above the panel in C and D) was established in the presence of G418. The cells were incubated for 72 h without (control) and with 15 µg/ml of C-29EA. Firefly or *Renilla* luciferase activity was measured by the method described in Materials and Methods and was normalized by the protein concentration. F/R: relative ratio of firefly luciferase activity to *Renilla* luciferase activity. F/R is presented as a percentage of the control condition. Error bars indicate standard deviation. The data represent three independent experiments.

Extract C-29EA had the most potent inhibitory activity against HCV replication. The viral replication (Fig. 1 B and 2 A) and viral proteins (Fig. 3 A and B) in replicon cell lines derived from genotype 1b strain Con1 and 2a strain JFH1 were decreased 72 h after treatment in a dose-dependent manner. HCV protein has been translated based on the positive-sense viral RNA in an IRES-dependent manner. The replicon RNA of HCV is composed of the 5′-UTR of HCV, indicator genes (a luciferase-fused drug-resistant gene), encephalomyocarditis virus (EMCV) IRES, the viral genes encoding complete or nonstructural proteins, and the 3′-UTR of HCV, in that order [26]. The replicon RNA replicated autonomously in several HCV replication-permissive cell lines derived from several hepatoma cell lines. Nonstructural proteins in replicon cells were polycistronically translated through EMCV IRES. The cap-dependent translated mRNA, including *Renilla* luciferase, EMCV IRES, and the firefly luciferase/neomycin-resistant gene, in that order, was constructed to examine the effect of the extract on EMCV-IRES-dependent translation (Fig. 3C). When the mRNA expression was transcribed by an EF promoter of the transfected plasmid in the presence of C-29EA, the ratio of firefly luciferase activity to *Renilla* luciferase activity was not changed (Fig. 3C). This suggested that treatment with C-29EA exhibited no effect on EMCV-IRES-dependent translation. Furthermore, treatment with C-29EA did not significantly affect the activity of HCV IRES that was used instead of EMCV IRES in the system described above (Fig. 3D). Thus, these results suggest that treatment with C-29EA exhibits no effect on EMCV- or HCV-IRES-dependent translation.

### Effect of C-29EA on the Interferon Signaling Pathway

**Figure 4:**
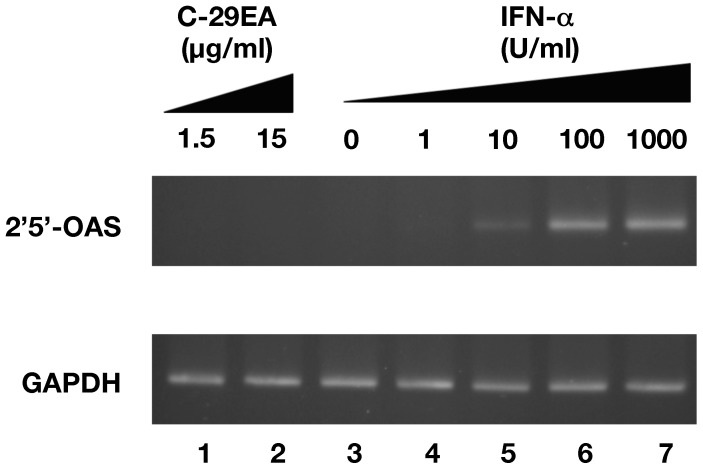
Effect of C-29EA on interferon signaling pathway. The Huh7 replicon cell line of genotype 1b was treated without (lane 3) or with 1, 10, 100, or 1000 U/mL interferon-alpha 2b (lanes 4–7), and 1.5 or 15 µg/ml C-29EA (lanes 1–2) for 48 h. Treatment with DMSO corresponds to ‘0′. The mRNAs of 2′, 5′-OAS, and GAPDH as an internal control were detected by RT-PCR. Error bars indicate standard deviation. The data represent three independent experiments.

It has been well known that HCV replication in cultured cells is potently inhibited by interferon [27], [28]. We examined whether or not treatment with C-29EA elicits an interferon-inducible gene from replicon cells. The replicon cells were treated with various concentrations of interferon-alpha 2b or 15 µg of C-29EA per milliliter. The treated cells were harvested at 72 h post-treatment. The interferon-inducible gene 2′, 5′-OAS, was induced with IFN-alpha 2b but not with a 10-times EC_50_ concentration of C-29EA (Fig. 4). These results suggest that the inhibitory effect of C-29EA on the replication of the HCV replicon is independent of the IFN signaling pathway.

### Effect of C-29EA on the NS3 Helicase Activity

**Figure 5:**
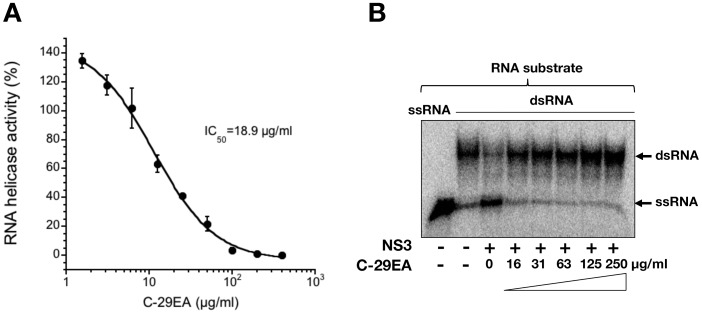
Effect of C-29EA on unwinding activity of NS3 helicase. (A) NS3 helicase activity was measured by PET assay. The reactions were carried out in the absence or presence of C-29EA. Helicase activity in the absence of C-29EA was defined as 100% helicase activity. Treatment with DMSO corresponds to ‘0′. The data are presented as the mean ± standard deviation for three replicates. (B) The unwinding activity of NS3 helicase was measured by an RNA unwinding assay using radioisotope-labeled RNA. The heat-denatured single-strand RNA (26-mer) and the partial duplex RNA substrate were applied to lanes 1 and 2, respectively. The duplex RNA was reacted with NS3 (300 nM) in the presence of C-29EA (lanes 4–9, 16–250 µg/ml). The resulting samples were subjected to native polyacrylamide gel electrophoresis. Treatment with DMSO corresponds to ‘0′.

We previously established an assay system for unwinding HCV activity based on photoinduced electron transfer (PET) [29], [30]. The fluorescent dye (BODIPY FL) is attached to the cytosine at the 5′-end of the fluorescent strand and quenched by the guanine base at the 3′-end of the complementary strand via PET. When helicase unwinds the double-strand RNA substrate, the fluorescence of the dye emits a bright light upon the release of the dye from the guanine base. The capture strand, which is complementary to the complementary strand, prevents the reannealing of the unwound duplex. Treatment with C-29EA inhibited the helicase activity in a dose-dependent manner, with an IC_50_ value of 18.9 µg/ml (Fig. 5A). We confirmed the effect of C-29EA on NS3 helicase unwinding activity by the RNA helicase assay using ^32^P-labeled double-stranded RNA (dsRNA) as a substrate. Treatment with C-29EA inhibited dsRNA dissociation at a concentration of 16 µg/ml and above (Fig. 5B).

**Figure 6:**
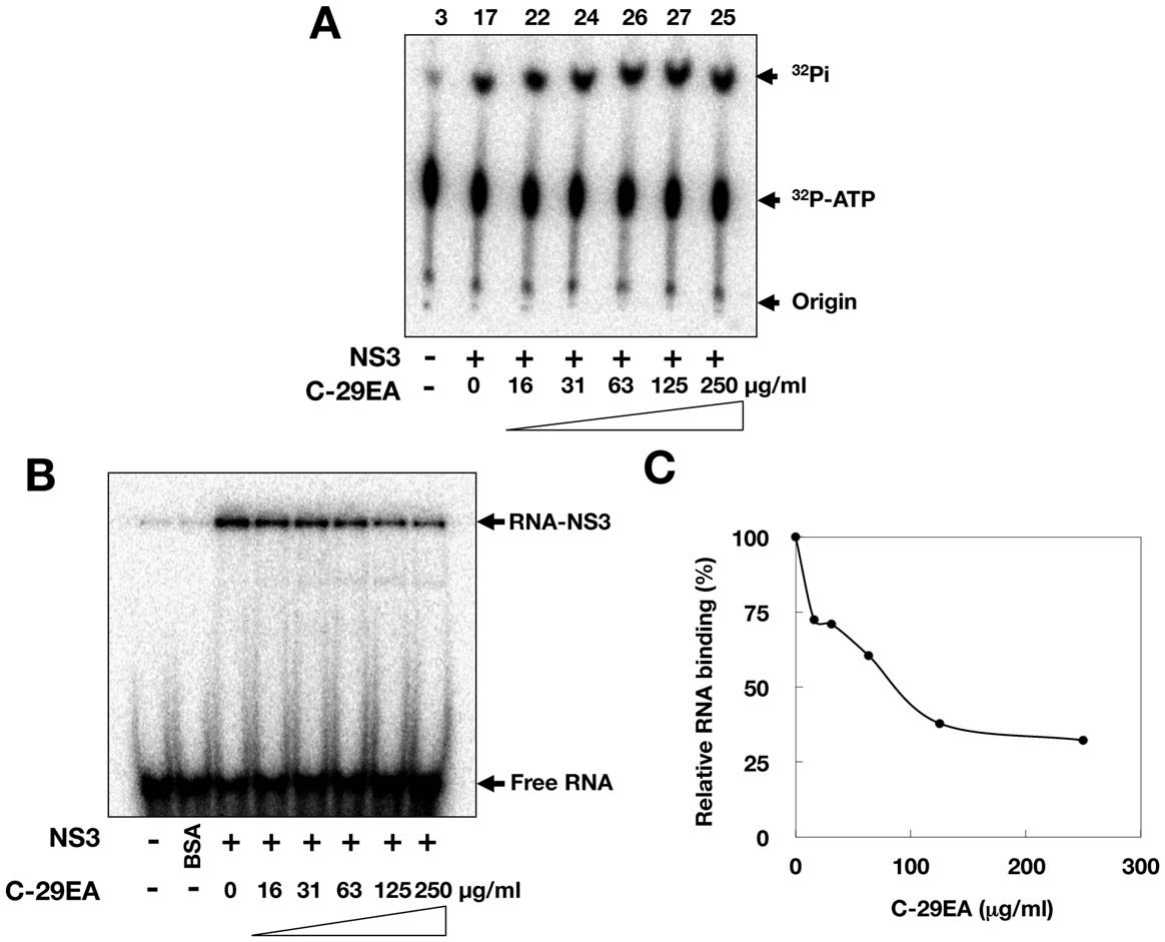
Effect of C-29EA on ATPase and RNA-binding activities of NS3 helicase. (A) The reaction mixtures were incubated with [γ-^32^P] ATP as described in Materials and Methods. The reaction mixtures were subjected to thin-layer chromatography. The start positions and migrated positions of ATP and free phosphoric acid are indicated as ‘Origin’, ‘^32^P-ATP’, and ‘^32^P-Pi’, respectively, on the right side of the figure. The data represent three independent experiments. Treatment with DMSO corresponds to ‘0′. (B) Gel mobility shift assay for RNA-binding activity of NS3 helicase. The reaction was carried out with 0.5 nM labeled ssRNA at the indicated concentrations of C-29EA or DMSO. The reaction mixture was subjected to gel mobility shift assay. (C) The relative RNA-binding ability was calculated with band densities in each lane and presented as a percentage of RNA-NS3 in the total density. The data represent three independent experiments. Treatment with DMSO corresponds to ‘0′.

The unwinding ability of HCV helicase depends on ATP binding, ATP hydrolysis, and RNA binding [30], [31]. We examined the effect of C-29EA on the ATPase activity of NS3. The ratio of free phosphate (^32^P-Pi) to ATP (^32^P-ATP) was determined in the presence of C-29EA. The reaction was carried out between 16 and 250 µg of C-29EA per milliliter. The ATPase activity of NS3 helicase was not inhibited (Fig. 6A), although the helicase activity was decreased to less than 20% in the presence of 50 µg of C-29EA per milliliter (Fig. 5A). Next, we examined the effect of C-29EA on the binding of NS3 helicase to single-strand RNA (ssRNA). A gel-mobility shift assay was employed to estimate the binding activity of NS3 to the 21-mer of ssRNA. The binding of NS3 to ssRNA was inhibited by C-29EA in a dose-dependent manner (Fig. 6 B and C). These results suggest that treatment with C-29EA inhibits the helicase activity of NS3 by suppressing RNA binding.

### Effect of C-29EA on NS3 Protease Activity

**Figure 7:**
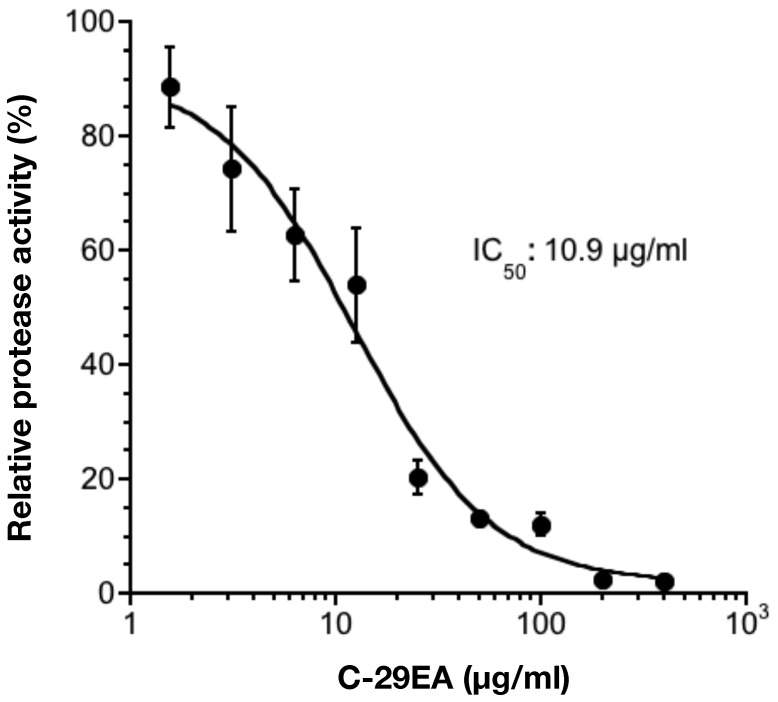
Effect of C-29EA on the activity of NS3 serine protease. NS3/4A serine protease was mixed with various concentrations of C-29EA or DMSO (0) in the reaction mixture and then incubated at 37°C for 120 min. The initial velocity at each concentration of C-29EA was calculated during 120 min reaction. The initial velocity in the absence of C-29EA was defined as 100% of relative protease activity. The data are presented as the mean ± standard deviation for three replicates.

Serine protease and helicase domains are respectively located on the N-terminal and C-terminal portions of NS3 [32]. Thus, we examined the effect of C-29EA on NS3 protease activity by using an NS3 protease assay based on FRET. NS3/4A serine protease was mixed with various concentrations of C-29EA. The initial velocity at each concentration of C-29EA was calculated during a 120 min reaction. The initial velocity in the absence of C-29EA represented 100% of relative protease activity. C-29EA decreased the serine protease activity in a dose-dependent manner (Fig. 7). The IC_50_ of C-29EA was 10.9 µg/ml, which is similar to the value estimated by helicase assay. These results suggest that C-29EA includes the compound(s) inhibiting the protease activity of NS3 in addition to the helicase activity.

### Combination Antiviral Activity of C-29EA and Interferon-alpha

**Figure 8:**
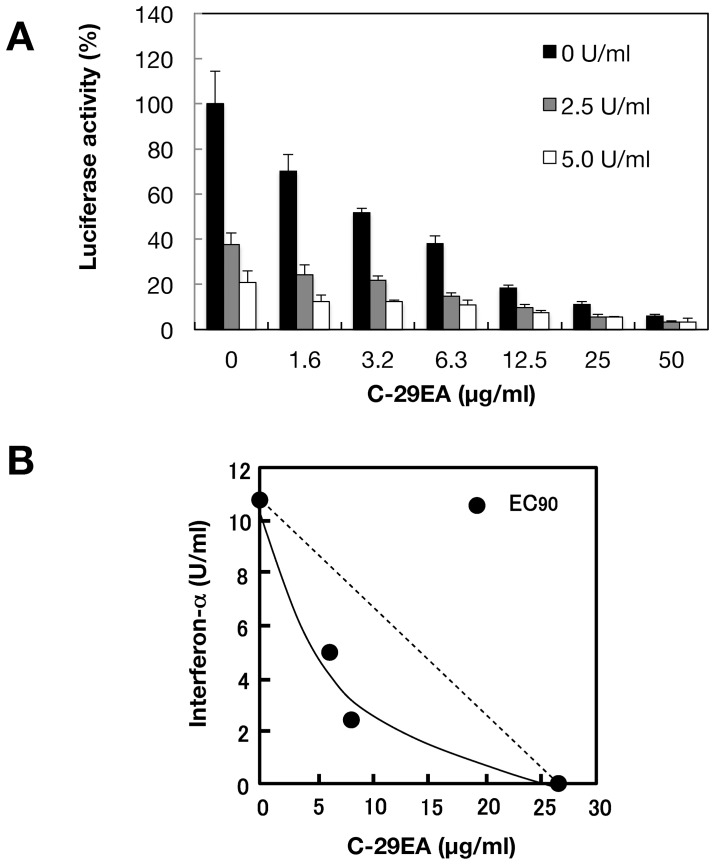
Effect of C-29EA on the antiviral activity of interferon-alpha. (A) The Huh7 cell line, including the subgenomic replicon RNA of genotype 1b strain Con1, was incubated in medium containing various concentrations of C-29EA or DMSO (0) in the presence or the absence of interferon-alpha. Luciferase assay were carried out as described in Materials and Methods. Error bars indicate standard deviation. The data represent three independent experiments. (B) Isobole plots of 90% inhibition of HCV replication. The broken line indicates the additive effect in the isobologram.

Treatment with C-29EA may potentiate inhibitory action of interferon-alpha, since it inhibited the protease and helicase activities of NS3 but not induce the interferon response as described above. Then, we examined effect of treatment using both interferon and C-29EA on HCV replication. The replication of replicon was decreased in the presence of C-29EA or interferon-alpha and further decreased by combination treatment using interferon-alpha and C-29EA (Fig. 8A). Furthermore, we employed the isobologram method [33] to determine whether antiviral effect of the combination treatment exhibits additive or synergistic. EC_90_ values of interferon-alpha and C-29EA were estimated at 10.7 U/ml and 26.4 µg/ml, respectively, in the absence of each other. EC_90_ values of C-29EA in the presence of 0, 2.5 and 5 U/ml interferon-alpha were plotted to generate an isobole. Figure 8B shows that the isobole exhibits concave curvilinear, representing synergy but not additivity. These results suggest that combination treatment of interferon-alpha and C-29EA exhibits synergistic inhibition of HCV replication.

## Discussion

Several natural products have been reported as anti-viral agents against HCV replication. Silbinin, epigallocatechin 3-gallate, and proanthocyanidins, which were prepared from milk thistle, green tea, and blueberry leaves, respectively, have exhibited inhibitory activity against HCV replication in cultured cells [34]–[37]. In our previous report, we identified manoalide as an anti-HCV agent from a marine sponge extract by high-throughput screening targeting NS3 helicase activity [38]. Manoalide inhibited ATPase, RNA binding, and NS3 helicase activity in enzymological assays. The EtOAc extract of the marine feather star also suppressed HCV replication in HCV replicon cell lines derived from genotype 1b, and it inhibited the RNA-binding activity but not the ATPase activity of NS3 helicase [30]. In this study, we screened 84 extracts of marine organisms for their ability to inhibit HCV replication in replicon cell lines and HCV cell culture system. Among these extracts, C-29EA, which was extracted from *Amphimedon* sp., most strongly inhibited HCV replication regardless of cytotoxicity. We previously reported that the EtOAc extract (SG1-23-1) of the feather star *Alloeocomatella polycladia* inhibited HCV replication with an EC_50_ of 22.9 to 44.2 µg/ml in HCV replicon cells derived from genotype 1b [30]. Treatment with C-29EA potently inhibited HCV replication with an EC_50_ of 1.5 µg/ml and with an SI of more than 33.3 in the replicon cell line derived from genotype 1b, regardless of cytotoxicity (Fig. 1B and Table 2). However, C-29EA exhibited an EC_50_ of 24.9 µg/ml in a replicon cell line derived from genotype 2a at a weaker level than in the replicon cell line derived from genotype 1b (Figs. 1 and 2), suggesting that the ability of C-29EA to suppress HCV replication is dependent on the viral genotype or strain.

HCV NS3 is well known to play a crucial role in viral replication through helicase and protease activities [5], [39]. The N-terminal third of NS3 is responsible for serine protease activity in order to process the C-terminal portion of polyprotein containing viral nonstructural proteins [32]. The remaining portion of NS3 exhibits ATPase and RNA-binding activities responsible for helicase activity, which is involved in unwinding double-stranded RNA during replication of genomic viral RNA [40]–[42]. A negative-strand RNA is synthesized based on a viral genome (positive strand) after viral particles in the infected cells are uncoated, and is then used itself as a template to synthesize a positive-stranded RNA, which is translated or packaged into viral particles. Thus, both helicase and protease activities of NS3 are critical for HCV replication and could be targeted for the development of antiviral agents against HCV.

NS3 helicase activity was inhibited by treatment with C-29EA in a dose-dependent manner with an IC_50_ of 18.9 µg/ml (Fig. 5A). RNA-binding activity, but not ATPase activity, was inhibited by treatment with C-29EA (Fig. 6). Treatment with C-29EA did not significantly affect the HCV-IRES activity and did not induce interferon-stimulated gene 2′,5′-OAS (Figs. 3 and 4). Furthermore, the serine protease activity of NS3 was inhibited by using C-29EA with an IC_50_ of 10.9 µg/ml (Fig. 7). These results suggest that *Amphimedon* sp. includes the unknown compound(s) that could suppress NS3 enzymatic activity to inhibit HCV replication. Although the mechanism by which treatment with C-29EA could inhibit HCV replication has not yet been revealed, the unknown compound(s) may be associated with the inhibition of NS3 protease and helicase, leading to the suppression of HCV replication. However, other effects of extract C-29EA on HCV replication could not be excluded in this study.

The compound 1-N, 4-N-bis [4-(1H-benzimidazol-2-yl)phenyl] benzene-1,4-dicarboxamide, which is designated as (BIP)_2_B, was reported to be a potent and selective inhibitor of HCV NS3 helicase [43]. This compound competitively decreases the binding ability of HCV NS3 helicase to nucleic acids. The compound (BIP)_2_B inhibited RNA-induced stimulation of ATPase, although it did not directly affect the ATP hydrolysis activity of NS3 helicase. Thus, (BIP)_2_B could not affect ATPase activity without RNA or with a high concentration of RNA. Treatment with C-29EA inhibited helicase activity and viral replication but not ATPase activity (Figs. 1B, 2, 5, and 6). This extract suppressed the binding of RNA to helicase but exhibited no suppression of ATPase by NS3 helicase. Thus, the inhibitory action of extract C-29EA seems different from that of (BIP)_2_B. The quinolone derivative QU663 was reported to inhibit the unwinding activity of NS3 helicase by binding to an RNA-binding groove irrespective of its own ATPase activity [44]. The compound QU663 may competitively bind the RNA-binding site of NS3 but not affect ATPase activity, resulting in the inhibition of unwinding activity. In this study, treatment with C-29EA inhibited the RNA-binding activities of NS3 helicase but did not affect ATPase activity (Fig. 6). Furthermore, treatment with C-29EA suppressed the viral replication of HCV in an HCV cell culture system derived from several virus strains (Figs. 1 and 2, Table 2). The mechanism of C-29EA on the inhibition of NS3 helicase may be similar to that of compound QU663.

It is unknown whether one or several molecules included in C-29EA are critical for the inhibition of protease and helicase activities. The serine protease NS3/4A is one of the viral factors targeted for development into antiviral agents. Improvements in HCV therapy over the past several years have resulted in FDA approval of telaprevir (VX-950) [15], [45] and boceprevir (SCH503034) [46], [47]. Several studies suggest that the activities of NS3/4A protease and helicase in the full-length molecule enhance each other [48], [49]. The NS3/4A protease has formed a complex with macrocyclic acylsulfonamide inhibitors [50], [51]. Schiering et al. recently reported the structure of full-length NS3/4A in complex with a macrocyclic acylsulfonamide protease inhibitor [52], although the structure of full-length HCV NS3/4A in complex with a protease inhibitor has not been reported. The inhibitor binds to the active site of the protease, while the P4-capping and P2 moieties of the inhibitor are exposed toward the helicase interface and interact with both protease and helicase residues [52]. An unknown compound included in C-29EA might interact with both protease and helicase domains of NS3 to inhibit their activities. However, our data in this study have not excluded the possibility that several compounds included in C-29EA are related to the inhibition of protease and helicase of NS3/4A.

In conclusion, we showed that the EtOAc extract from *Amphimedon* sp. significantly inhibits HCV replication by suppressing viral helicase and protease activities. The purification of an inhibitory compound from the extract of *Amphimedon* sp. will be necessary in order to improve its efficacy by chemical modification.

## Materials and Methods

### Preparation of Extracts from Marine Organisms

All marine organisms used in this study were hand-collected by scuba diving off islands in Okinawa Prefecture, Japan. No specific permits were required for the described field studies. We do not have to obtain a local government permit to collect invertebrates except for stony corals and marine organisms for fisheries, which we did not collect in this study. The areas where we collected are not privately-owned or protected in any way. We did not collect any invertebrates listed in the red data book issued by Ministry of Environment, Japan. The sponges, tunicates, and soft corals used in this study are not listed at all. Hence, no specific permits are required for this collection in the same way as the previous report of Aratake et al. [53].

The sponge from which C-29EA was extracted was identified as *Amphimedon* sp. and deposited at Naturalis under the code RMNH POR 6100. Each specimen was soaked in acetone. The acetone-extract fraction prepared from each specimen was concentrated. The resulting material was fractionated as an EtOAc- and water-soluble fraction. The water-soluble fraction was dried up and solubilized in MeOH. The EtOAc- and the MeOH-soluble fractions were used for screening. All samples were dried and then solubilized in dimethyl sulfoxide (DMSO) before testing.

### Cell Lines and Virus

The following Huh-7-derived cell lines used in this study were maintained in Dulbecco’s modified Eagle’s medium containing 10% fetal calf serum and 0.5 mg/ml G418. The Lunet/Con1 LUN Sb #26 cell line, which harbors the subgenomic replicon RNA of the Con1 strain (genotype 1b), was kindly provided by Ralf Bartenschlager [26]. Huh7/ORN3-5B #24 cell line, which harbors the subgenomic replicon RNA of the O strain (genotype 1b) was reported previously [54] and used for screening in this study (Table 1). HCV replicon cell line derived from genotype 2a strain JFH1 was described previously [55]. The surviving cells were infected with the JFH-1 virus at a multiplicity of infection (moi) of 0.05. The viral RNA derived from the plasmid pJFH1 was transcribed and introduced into Huh7OK1 cells according to the method of Wakita et al. [56]. The infectivity of the JFH1 strain was determined by a focus-forming assay [56].

### Quantitative Reverse-transcription PCR (qRT-PCR) and Estimation of Core Protein

The estimation of viral RNA genome was carried out by the method described previously [57] with slight modification. Total RNAs were prepared from cells and culture supernatants by using an RNeasy mini kit (QIAGEN, Tokyo, Japan) and QIAamp Viral RNA mini kit (QIAGEN), respectively. First-strand cDNA was synthesized by using a high capacity cDNA reverse transcription kit (Applied Biosystems, Carlsbad, CA, USA) with random primers. Each cDNA was estimated by using Platinum SYBR Green qPCR SuperMix UDG (Invitrogen, Carlsbad, CA, USA) according to the manufacturer’s protocol. Fluorescent signals of SYBR Green were analyzed by using an ABI PRISM 7000 (Applied Biosystems). The HCV internal ribosomal entry site (IRES) region was amplified using the primer pair 5′- GAGTGTCGTGCAGCCTCCA -3′ and 5′- CACTCGCAAGCACCCTATCA -3′. Expression of HCV core protein was determined by an enzyme-linked immunosorbent assay (ELISA) as described previously [57].

### Determination of Luciferase Activity and Cytotoxicity in HCV Replicon Cells

HCV replicon cells were seeded at 2×10^4^ cells per well in a 48-well plate 24 h before treatment. C-29EA was added to the culture medium at various concentrations. The treated cells were harvested 72 h post-treatment and lysed in cell culture lysis reagent (Promega, Madison, WI, USA) or *Renilla* luciferase assay lysis buffer (Promega). Luciferase activity in the harvested cells was estimated with a luciferase assay system (Promega) or a *Renilla* luciferase assay system (Promega). The resulting luminescence was detected by the Luminescencer-JNR AB-2100 (ATTO, Tokyo, Japan) and corresponded to the expression level of the HCV replicon. Cell viability was measured by a dimethylthiazol carboxymethoxy-phenylsulfophenyl tetrazolium (MTS) assay using a CellTiter 96 aqueous one-solution cell proliferation assay kit (Promega).

### Effects on Activities of Internal Ribosome Entry Site (IRES)

Huh7 cells were transfected with pEF.Rluc.HCV.IRES.Feo or pEF.Rluc.EMCV.IRES.Feo and then were established in medium containing 0.25 mg/ml G418, as described previously [58]. These cell lines were seeded at 2×10^4^ cells per well in a 48-well plate 24 h before treatment, treated with 15 µg/ml extract C-29EA, and then harvested at 72 h post-treatment. The firefly luciferase activities were measured with a luciferase assay system (Promega). The total protein concentration was measured using the BCA Protein Assay Reagent Kit (Thermo Scientific, Rockford, IL, USA) to normalize luciferase activity.

### Western Blotting and Reverse-transcription Polymerase Chain Reaction (RT-PCR)

Western blotting was carried out by a method described previously [30]. The antibodies to NS3 (clone 8G-2, mouse monoclonal, Abcam, Cambridge, UK), NS5A (clone 256-A, mouse monoclonal, ViroGen, Watertown, MA, USA), and beta-actin were purchased from Cell Signaling Technology (rabbit polyclonal, Danvers, MA, USA) and were used as the primary antibodies in this study. RT-PCR was carried out by a method described previously [30], [58].

### Assays for RNA Helicase, ATPase, and RNA-binding Activities

A continuous fluorescence assay based on photoinduced electron transfer (PET) was described previously [29] and was slightly modified with regard to the reaction mixture [30]. The NS3 RNA unwinding assay was carried out by the method of Gallinari et al. [59] with slight modifications [30]. NS3 ATPase activity was determined by the method of Gallinari et al. [59] with slight modifications [30]. RNA binding to NS3 helicase was analyzed by a gel mobility shift assay [30], [31]. The gene encoding NS3 helicase was amplified from the viral genome of genotype 1b and was introduced into a plasmid for the expression of a recombinant protein [38], [60]. The radioactive band was visualized with the Image Reader FLA-9000 and quantified by Multi Gauge V 3.11 software.

### NS3 Protease Assay

The fluorescence NS3 serine protease assay based on fluorescence resonance energy transfer (FRET) was carried out by the modified method using the SensoLyte™ 520 HCV protease assay kit (AnaSpec, Fremont, CA, USA). In brief, NS3 protein with a two-fold excess of NS4A cofactor peptide (Pep4AK) was prepared in 1× assay buffer provided with the kit. HCV NS3/4A protease was mixed with increasing concentrations of C-29EA and incubated at 37°C for 15 min. The reaction was started by adding the 5-FAM/QXL 520 substrate to the reaction mixture containing 180 nM HCV NS3/4A protease and various concentrations (0–400 µg/ml) of C-29EA. The resulting mixture (20 µl) was incubated at 37°C for 120 min using a LightCycler 1.5 (Roche Diagnostics, Basel, Switzerland). The fluorescence intensity was recorded every minute for 120 min. The NS3 serine protease activity was calculated as the initial reaction velocity and presented as a percentage of relative activity to that of the control examined with DMSO solvent but not C-29EA, in the same way as described in the fluorescence helicase assay [29].

### Analysis of Drug-drug Interaction

The effects of drug combinations were evaluated using the isobologram method [33]. Various doses of C-29EA and interferon-alpha on 90% inhibition of HCV replication were combined to generate an isoeffect curve (isobole) to determine drug–drug interaction. Concave, linear, and convex curves exhibit synergy, additivity, and antagonism, respectively.

### Statistical Analysis

The results are expressed as the mean ± standard deviation. The significance of differences in the means was determined by Student’s *t*-test.
